# Sir Isaac Newton Stranger in a Strange Land

**DOI:** 10.3390/e22111204

**Published:** 2020-10-25

**Authors:** Bruce J. West

**Affiliations:** Office of the Director Army Research, Research Triangle Park, NC 27709, USA; brucejwest213@gmail.com

**Keywords:** complexity, chaos, fractional calculus, subordination

## Abstract

The theme of this essay is that the time of dominance of Newton’s world view in science is drawing to a close. The harbinger of its demise was the work of Poincaré on the three-body problem and its culmination into what is now called chaos theory. The signature of chaos is the sensitive dependence on initial conditions resulting in the unpredictability of single particle trajectories. Classical determinism has become increasingly rare with the advent of chaos, being replaced by erratic stochastic processes. However, even the probability calculus could not withstand the non-Newtonian assault from the social and life sciences. The ordinary partial differential equations that traditionally determined the evolution of probability density functions (PDFs) in phase space are replaced with their fractional counterparts. Allometry relation is proven to result from a system’s complexity using exact solutions for the PDF of the Fractional Kinetic Theory (FKT). Complexity theory is shown to be incompatible with Newton’s unquestioning reliance on an absolute space and time upon which he built his discrete calculus.

## 1. Introduction

Three centuries ago, Newton transformed Natural Philosophy into today’s Science by focusing on change and mathematical quantification and he did so in a way that resonated with the scientific community of his day. His arguments appeared to be geometric in character, and nowhere in the *Principia* do you find explicit reference to fluxions, or to differentials. What Newton did was reveal the entailments of the calculus and convince generations of scientists of the value of their focusing on how physical objects change their location in time. Some contemporary mathematicians of his generation recognized what he had done, but their number can be counted on one hand, and their comments are primarily of historical interest.

Fast forward to today, where modern science, from Anatomy to Zoology, is seen to have absorbed the transformational effect of Newton’s contribution to how we quantitatively and qualitatively understand the world, the fundamental importance of motion. However, it has occurred to a number of the more philosophically attuned contemporary scientists that we are now at another point of transition, where the implications of complexity, memory, and uncertainty have revealed themselves to be barriers to our future understanding of our technological society. The fractional calculus (FC) has emerged from the shadows as a way of taming these three disrupters with a methodology capable of analytically smoothing their singular natures.

If Sir Isaac Newton were reincarnated into the modern world would he again achieve scientific greatness using his prodigious intellect? Of course we cannot know the answer to this counterfactual, but what we can determine is whether his fundamental assumptions upon which the physical laws of analytic mechanics are based remain valid in the today’s world of complexity science. Whether or not Newton would remain a stranger in this strange land of today’s science is the question we seek to answer in this essay. Not literally, of course, but more to the point whether the fundamental assumptions on which his mechanics is based can be sufficiently modified to be compatible with the mathematics found necessary to describe today’s complex phenomena, without being distorted to the point of being abandoned. Can Newton’s view of the world be made compatible with the FC?

The FC moldered in the mathematical backwaters for over 300 years. Since the time of Newton it was mostly ignored by the social, physical, and life scientists, intermittently emerging from the shadows of formalism with an application. Historically, the community of international physical scientists saw no need for a new calculus, or if occasionally seeing the need thought it not worthy of acknowledgment. The community agreed that the ordinary differential calculus of Newton and Leibniz, along with the analytic functions entailed by solving the equations resulting from Newton’s force law, are all that is required to provide a scientific description of the macroscopic physical world.

In his *Mathematical Principles of Natural Philosophy* [1], Newton introduced mathematics into the study of *Natural Philosophy*. He argued the need for quantification of scientific knowledge through the introduction of mathematics in the form of *fluxions* and thereby changed the historical goal of natural philosophy from that of wisdom to that of knowledge. This new term fluxion does not appear anywhere in the *Principles*, but scholars have found numerous geometric arguments, which, in fact, were in all probability based on limits in which Newton, no doubt, had differentials in the back of his mind. The Marquis de l’Hôpital commented that Newton’s *magnum opus* was “a book dense with the theory and application of the infinitesimal calculus”; an observation also made in modern times by Whiteside [2].

Along with mathematics, Newton also introduced a number of definitions that determined how scientists were to understand his vision of the physical world for the next few hundred years. We do not quote his definitions of such well-known things as inertia and force here, but instead we record the notions of space and time that he believed were the accepted understanding of their meanings as explained in his first scholium (A scholium is a marginal note or explanatory comment made by a scholar), which are [1] as follows.

I Absolute, time, and mathematical time, of itself, and from its own nature, flows equably without relation to anything external, and by another name is called duration: relative, apparent, and common time, is some sensible and external (whether accurate or unequable) measure of duration by the means of motion, which is commonly used instead of true time; such as an hour, a day, a month, a year.
II Absolute space, in its own nature, without relation to anything external, remains always similar and immovable. Relative space is some movable dimension or measure of the absolute space; which our senses determine by its position to bodies; and which is commonly taken for immovable space; such is the dimension of subterraneous, an aerial, or celestial space, determined by its position in respect of the earth. Absolute and relative space are the same in figure and magnitude; but they do not remain always numerically the same. For if the earth, for instance, moves, a space of our air, which relatively and in respect of the earth remains always the same, will at one time be one part of the absolute space into which the air passes; at another time it will be another part of the same, and so, absolutely understood, it will be continually changed.

Newton’s understanding of these two notions of the absolute are what enabled him to invent fluxions and introduce motion as the basis for his new physics. Of course, the mathematically awkward discrete notation of fluxions was subsequently elbowed out of history by the user-friendly notation of Leibniz, which became known as the differential calculus. The differential calculus enabled subsequent generations of scientists to describe the motion of particles in terms of continuous single particle trajectories in space and time. The differential calculus fills literally thousands of mathematics/physics text books; all assuming that I and II codify the real world and are taught to eager students and novitiate scientists throughout the world. Herein, we argue for a mathematics that provides a logical framework for understanding the more complex world of the Information Age, in which I and II must be applied with extreme caution, if at all.

The increase in sensitivity of diagnostic tools, advances in data processing techniques, and expanding computational capabilities have all contributed to the broadening of science in ways that have brought many phenomena from borderline interest to center stage. These curious complex processes are now cataloged under the heading of non-integer scaling phenomena. An understanding of the fundamental dynamics underlying such scaling requires a new mathematical perspective, such as that obtained using the dynamics described by non-integer (fractional) operators and such descriptions ushered in the sunset for much of what remains of Newton’s world view.

Much of what is written in this Introduction will be familiar to those with a background in physics, even if the organization of the material is not. However the reasons why classical physics fails to explain a given complex phenomena remains a mystery to those without such a background as well as to many who do. Therefore, we express the purpose of this paper in the form of a hypothesis and present arguments in support of the Complexity Hypothesis (CH):

Complex phenomena entailing description by chaos theory, fractional Kinetic Theory, or the fractional calculus in general, are incompatible with the ordinary calculus and consequently are incompatible with Newtonian Physics.

### 1.1. The Demise of Newton’s World View?

The evidence is all around us that the domain of application of Newton’s view of the physical world is contracting dramatically. His view was reluctantly contracted with the introduction of quantum mechanics along with relativity over a century ago. However, physicists took consolation in the fact that the dynamic predictions of the very fast, the very large, and the very small, all reduce to those of Newton in the appropriate limits. For special relativity, the dramatic changes in time occur as the speed of light is approached [3]; for general relativity, space curves in the neighborhood of a large mass [4]; and for quantum phenomena, the correspondence principle associated with the size of Planck’s constant insures the quantized nature of energy is lost at large quantum numbers and energy is continuous on the scale we live our lives [5]. However, the more recent constrictions produced by chaotic dynamics is different; so much so that once made, there is no limit in which the view of Newton can reemerge. This requires more explanation, as the inappropriate application of the differential calculus to describe the dynamics of strongly nonlinear phenomena often yields misleading results. In the author’s view, one such misinterpretation arose in support of the political interpretation of climate change.

It should be evident that the rubric *climate change* provides an example of such a misapplication of the nonlinear hydrodynamic partial differential equations that purport to describe the internal motion of the earth’s atmosphere involving the multiple interactions with the earth’s temperature field, solar radiation, cloud cover, and all the rest. Climate change is not just a problem in Newtonian physics, because if it were we would have the answer to the problem in hand, which some few scientists believe we do. I say this with full appreciation for the criticism such a statement will draw, from both the believers in climate change and the sceptics who do not. Let me be absolutely clear in stating that I believe in climate change, but belief is the wrong word. Climate change is a scientific fact not a matter of faith or belief. What I am skeptical about concerns the quasi-scientific arguments used in the political arena that assign causality of that change to human activity followed by the assertion that climate change can be significantly influenced by political action.

I came to this conclusion, not through a “eureka” moment, or flash of insight, but more through the weight of evidence drawn from my own scientific research. I even coauthored a book about it [6] with a colleague who was then a post-doctoral researcher of mine. Our book addressed climate change as a problem in physics and was greeted with a yawn from the scientific community. It was the last scientific contribution I made to that debate and the science has not moved significantly since its publication. My epiphany was that those who successfully communicate technically difficult ideas tell a story. Thus, I have decided to populate this essay with a sequence of technically-based stories. Each one lending additional support to the CH. The first story concerns chaos theory and some of what that entails.

### 1.2. Chaos Theory

The chaos story begins in the middle nineteenth century with Oscar II, the King of Sweden and Norway, and his concern over how long the Earth will survive. More pointedly, he wondered whether the solar system was stable. Could one expect the moon to spiral out of its orbit and crash into the Earth? Would the Earth break from its timeless trajectory and collide with the Sun? Let me stop here and say this is the beginning of the somewhat romanticized historical account of how chaos came into being that I learned when I was first introduced to the “three-body problem” as a freshly mined minted physics PhD in 1970. The actual historical account is a bit more banal, but not much.

Oscar II had done well in mathematics while a university student and had grown into an active patron of the subject [7], so his sponsorship of a prize in mathematics, unrelated to any particular institution was not surprising. Mittag-Leffler, who was then the editor of the Swedish journal *Acta*, made the original announcement of the King’s mathematics competition, in the science magazine *Nature*. In that announcement Mittag-Leffler listed four categories to which international scientists could submit contributions. The category concerning the stability of the solar system was written in the following arcane way [7].

(1) A system being given of a number whatever of particles attracting one another mutually according to Newton’s law, it is proposed, on the assumption that there never takes place an impact of two particles to expand the coordinates of each particle in a series proceeding according to some known functions of time and converging uniformly for any space of time.

The committee that evaluated the submissions to the competition consisted of, along with Mittag-Leffler, two other giants of nineteenth century mathematics, Hermite and Weierstrass. To avoid any possibility of bias the entrants and their submissions remained anonymous until the winner was selected, at which time the name was to be published in *Acta*. Out of a field of 12 entrants, the committee selected Henri Poincaré, who had responded to question (1). He extended the analysis of the solvable two-body problem to the addition of one additional body, which was much less massive than the other two. Poincaré proved that the solution to Newton’s dynamic equations for his restricted three-body problem could not have a simple analytic form. His published proof entailed the invention of new mathematics, the implications of which have kept the best mathematician in the world actively engaged for over a century.

In reviewing the prize-winning memoire for publication in *Acta*, a referee pointed out an error in the manuscript. Part of the drama associated with publishing the final version of the paper concerned the secrecy surrounding that error. Correcting this error entailed a major rewrite, which took Poincaré nearly a year to complete. In composing the revision, he conceived of and implemented in the manuscript the idea of a homoclinic point [7], which is the basis of our understanding of what today goes by the popular name of chaos theory. In short, he introduced the *Three-Body Problem* to the scientific community as being of fundamental importance and proved that the elliptic orbits of the two-body problem were replaced by orbits in the restricted three-body problem that resembled nothing so much as a plate of spaghetti. A single strand of entangled spaghetti was the convoluted trajectory of the third body and the asymptotic position of the body along that trajectory at any time was unpredictable. Today we call such trajectories fractals [8].

Sir James Lighthill, on the three-hundred-year anniversary of the communication of Newton’s *Principia* to the Royal Society, and while he was president of the *International Union of Theoretical and Applied Mechanics*, published the paper *The recently recognized failure of predictability in Newtonian dynamics* [9]. In this paper, Lighthill traces the history of mechanics from Tycho Brahe collecting astronomical data as a court astronomer, through Poincaré’s proof of the limited predictability horizon of Newton’s law of the dynamics of mechanical systems. To put this in a proper perspective let us use Lighthill’s words:

We are all deeply conscious today that the enthusiasm of our forebears for the marvelous achievements of Newtonian mechanics led them to make generalizations in this area of predictability which, indeed, we may have generally tended to believe before 1960, but which we now recognize were false. We collectively wish to apologize for having misled the general educated public by spreading ideas about determinism of systems satisfying Newton’s laws of motion that, after 1960, were to be proved incorrect…

This reluctant indictment of the Newtonian system of nonlinear partial differential equations that describe how the radiation from the sun is absorbed by the earth’s atmosphere and redistributed around the globe has to the best of my knowledge never been explicitly refuted. This is not unexpected as Sir James was the scientific leader in the area of applied mathematics involving those same equations for over thirty years. If the unpredictability of coupled systems of nonlinear differential equation were expressed as a theorem, then one can draw a corollary regarding the nature of the computer simulations based on those same equations. The reader is free to infer from these remarks if Newton’s view is truly dead or whether it is just confined to an ever decreasing domain of analytic application.

What we can conclude with certainty is that Newton’s force law typically breaks down when the system being analyzed is not linear and the equations of motion are nonlinear. Such equations typically do not have analytic solutions, their solutions are generically chaotic [10,11]. As scientists, this loss of predictability, which is the foundation of the physical sciences, ought to be our greatest concern, or at least the mathematical foundation of all our physical models, the differential calculus, ought to be the focus of our concern.

It is worth mentioning that in his philosophical writings Poincaré recognized that his mathematical analysis entailed the loss of predictability and the existence of a new kind of chance [12]:

A very slight cause, which escapes us, determines a considerable effect which we can not help seeing, and then we say this effect is due to chance. If we could know exactly the laws of nature and the situation of the universe at the initial instant, we should be able to predict exactly the situation of this same universe at a subsequent instant. But even when the natural laws should have no further secret for us, we could know the initial situation only *approximately*. If that permits us to foresee the subsequent situation *with the same degree of approximation,* this is all we require, we say the phenomenon has been predicted, that it is ruled by laws. But this is not always the case: it may happen that slight differences in the initial conditions produce very great differences in the final phenomena: a slight error in the former would make an enormous error in the latter. Predication become impossible and we have the fortuitous phenomenon.

For over a century, some of the world’s leading mathematicians have been working on what on what might be a proper replacement for, or extension of, Newton’s physics. They typically begin with the notion that a conservative nonlinear dynamical system with three or more degrees-of-freedom is chaotic [13], which means that its dependence on initial conditions is so sensitive that an infinitesimal change in the initial state will produce a trajectory that exponentially diverges from the trajectory predicted by the original state. Such an exponential separation of trajectories means that the perturbed state is unstable in the sense that its asymptotic location cannot be predicted from the initial state.

The work that Lighthill was alluding to in his remarks quoted earlier were those of the meteorologist Ed Lorenz, whose ground breaking paper opened the world of fluid dynamics to the importance of chaos [14], and ended dreams of long-term weather forecasting. Those that have considered chaos as a possible obstacle to climate forecasting as well, treat it in much the same way that the nineteen century physicists Maxwell and Boltzmann treated many-body effects to produce Kinetic Theory. Only now the modern climate physicist examines large-scale computer simulations of the earth’s atmosphere as having random fluctuations around the average dynamical behavior of the atmosphere’s velocity field and temperature. The established procedure is to carry out a large number of computer simulations, all starting from the “same state”, and from them construct an ensemble of atmospheres with which to calculate the average dynamics of the interesting physical quantities.

The general impression in the meteorology community is that such ensemble averages ought to be sufficient to smooth out the influence of chaotic trajectories and thereby provide the appropriate phase space probability density function in the kinetic theory sense. The problem with the approach is when one actually attempts to average over an ensemble of chaotic trajectories the integer moments diverge leaving the coefficients ill-defined in the kinetic theory of Maxwell and Boltzmann. Here again we find a need for a new kind of mathematics and the fractional calculus comes to the rescue, providing a fractional Kinetic Theory (FKT).

In Section 2, we generalize the traditional phase space partial differential equations for the probability density function (PDF) to the fractional calculus. This is done by averaging over an ensemble of chaotic trajectories, and following the mathematical arguments of Zaslavsky [15] create a FKT. The solution to a simple fractional diffusion equation is shown to have a generic analytic form.

### 1.3. Allometry Relations

**Figure 1:**
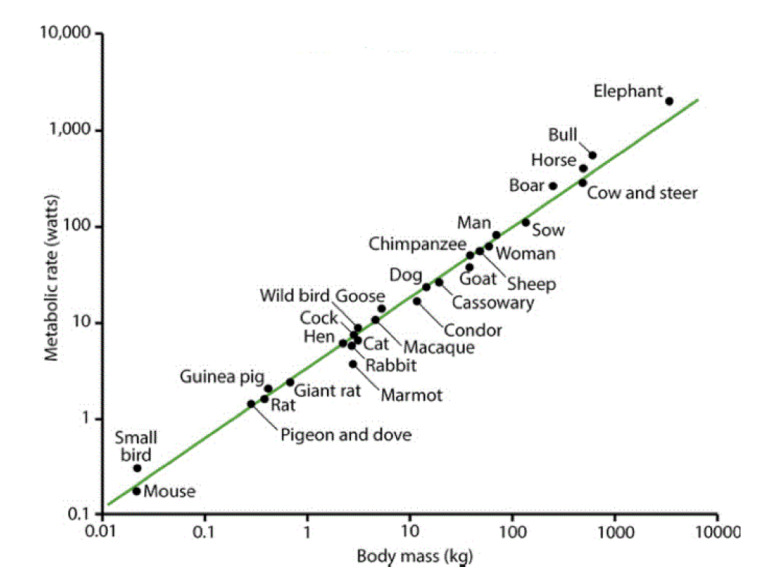
The mouse-to-elephant curve. The average metabolic rates of mammals and birds are plotted versus the average body weight (TBM) on log-log graph paper covering five orders of magnitude in size. The solid line segment is the best linear regression to the data from Schmidt-Neilson [18] with permission.

Scientists believed that phenomena whose dynamic description is the result of using non-integer operators, such as fractional derivatives, were interesting curiosities, but lay outside the mainstream of science. Even such empirical laws as allometry relations (ARs), in which the functionality of a system is related to a non-integer power of the system’s size, were thought to have causal relations, with traditional differential dynamic descriptions [16,17,18]. Perhaps the most famous allometry relation is that between the average metabolic rates of mammals and their average total body masses (TBMs) as depicted by the “mouse-to-elephant” curve in Figure 1. In this figure, the solid curve is a fit to data by a power-law relation of the form

(1) $$\langle Y\rangle =a{\langle X\rangle }^{b},$$

which is a straight line on log-log graph paper with slope b:

(2) $$\log\langle Y\rangle =\log a+b\log\langle X\rangle .$$

The functionality of the system *Y*, here the average metabolic rate is denoted by $\langle Y\rangle$ and the size of the system *X*, here the average TBM is denoted by $\langle X\rangle$. Note that the brackets here denote the empirical averaging process.

Historically such ARs were explained using biophysical arguments, for example, Sarrus and Rameaux [17,18] used simple geometrical arguments for heat transfer. They assumed the heat generated by a body is proportional to its volume and the heat is lost at the body’s surface and is proportional to surface area. The balance between the two suggested that the allometry parameter is given by the ratio of dimensions to be 2/3, which does not fit the data very well. The empirical value of the allometry parameter is b≈0.74, which was subsequently accounted for by using fractal scaling arguments [19]. A statistical technique based on the fractional calculus was developed in [20] to explain the averaging brackets in Equation (1), which in due course we use herein as an exemplar of complexity in the fractal statistics of physiological phenomena.

In Section 3, selected applications of the FC are presented with the intent of persuading the reader that as systems become more complex the value of the ordinary differential calculus to describe their behavior increasingly diminishes, until it is eventually nearly lost altogether. The analytic PDF that solves the simple FKT problem is shown to explain the empirical AR using a complexity-based arguments.

### 1.4. Another Time

The willingness of his contemporaries to accept Newton’s view of time flowing as an uninterrupted featureless stream is understandable. However, the reluctance of physicists to directly challenge Newton’s view of time outside extreme conditions in the physical sciences is unclear. This reluctance is not evident in psychology where everything we see, smell, taste and otherwise experience is in a continuous state of change. Consequently, the changes in the physical world are not experienced uniformly, which is another way of saying that there is an objective time associated with the physical and a subjective time associated with the psychological world. The physical scientists dismissed subjective time out of hand, prior to Einstein, but even after relatively the experiential time they accepted was considered to be a local physical time.

Here, we follow the discussion of Turalska and West [21]. The idea of different clocks telling different times arises naturally in physics; the linear transformation of Lorentz in relativistic physics being a familiar example. However, we are interested in the notion of multiple clocks in the biological and social sciences wherein they have begun distinguishing between cell-specific and organ-specific clocks in biology and person-specific and group-specific clocks in sociology [22]. Of course, the distinction between subjective and objective time dates back to the empirical Weber–Fechner Law [23] in the latter half of the nineteenth century.

While the global behavior of an organ, say the heart, might be characterized by apparently periodic cycles, the activity of single neurons demonstrate burstiness and noise. In a similar way people in a social group operate according to their individual schedules, not always performing particular actions in the same global time frame. Consequently, because of the stochastic behavior of one or both clocks, a probabilistic transformation between times is often necessary. An example of such a transformation is given by the subordination procedure.

Insight into the subordination procedure is provided if we begin by defining two clocks that operationalize time in two distinct ways. The ticking of the first clock records a subjective or operational discrete time *n*, which measures an individual’s time T(n). The ticking of the second clock records the objective or chronological time *t*, which measures the social time T(t) upon which a society of individuals agree. If each tick of the discrete clock *n* is considered to be an event, the relation between operational and chronological time is given by the waiting time PDF of those events in chronological time ψ(t). Assuming a renewal property for events, as given by a chain condition (convolution) from renewal theory in Section 2.1, one can relate operational to chronological time [21]:

(3) $$\langle T(t)\rangle =\overset{\infty }{\sum_{n=0}}\underset{0}{\int^{t}}\Psi \left(t-t^{\prime }\right)\psi_{n}\left(t^{\prime }\right)T\left(n\right)dt^{\prime }$$

Every tick of the operational clock is an event, which in the chronological time occurs at time intervals drawn from the renewal waitin -time PDF. This randomness entails the sum over all events and the result is an average over many realizations of the transformation. The last of the *n* events occurs at time $t^{\prime }$ and the survival probability $\Psi \left(t-t^{\prime }\right)$ insures that no further event occurs before the time t.

**Figure 2:**
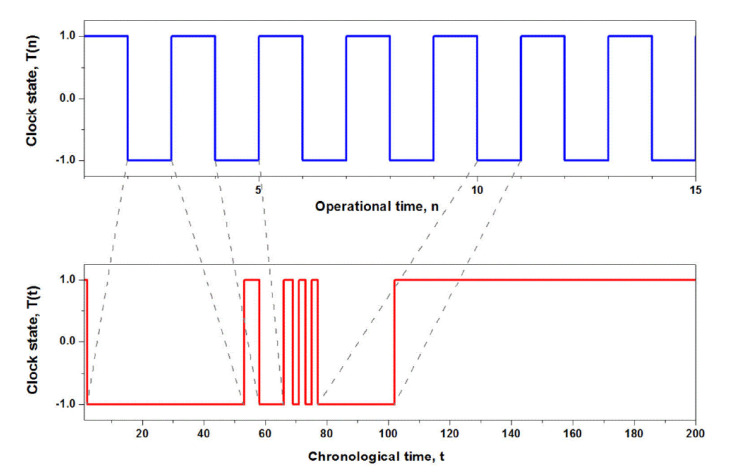
The upper curve is the regular transition between the two states of the individual in operational time. The lower curve is the subordination of the transition times to an IPL PDF to obtain chronological time.

For example, consider the behavior of a two-state operational clock, whose evolution is depicted in Figure 2, where the clock switches back and forth (tick tock) between its two states at equal time intervals. However, in chronological time this regular behavior is significantly distorted as seen in the figure. The time transformation was taken to be an inverse power law (IPL) waiting time PDF $\psi \left(t\right)$. Thus, a single time step in the operational time corresponds to a random time interval being drawn from ψ(t) in chronological time. The tail of the IPL PDF leads to especially strong distortions of the operational time trajectory, as there exist a non-zero probability of drawing very large time intervals between events. However, as the transformation between the operational and chronological time scales involves a random process, one needs to consider infinitely many trajectories in the chronological time, which leads to the average behavior of the clock in the chronological time denoted in Equation (3) by brackets.

Newton’s view of homogeneous isotropic time is shown to be incompatible with multiple phenomena in the social and life sciences in Section 3.2 using subordination theory. In that section the disciplines of biophysics, psychophysics, and sociophysics, to the degree they have adopted the Newtonian viewpoint, are shown to be misleading. The complexity of these disciplines require a new calculus to describe their dynamics.

In Section 3.2, we establish a direct link between subordination theory and the FC. This has been done in the literature in a number of different ways. In Section 2, we show how the probability calculus can be generalized to the FC in order to include temporal memory and spatial heterogeneity with probability theory.

What is entailed by the results presented herein is discussed in Section 5 and some conclusions are drawn.

## 2. Fractional Kinetic Theory

Zaslavsky [24] considered chaotic dynamics, as a physical phenomenon, to be a bridge spanning the gap between deterministic and stochastic dynamic systems. The dynamic states in the first case are described by regular functions and in the second by kinetic or other probabilistic equations. He developed the mathematics for the fractional kinetics corresponding to chaotic dynamics that is intermediate between completely regular (integrable) and completely random cases. The kinetics become “strange” because some moments of the PDF are infinite and the Onsager Principle is violated in that it takes infinitely long for fluctuations to relax back to the equilibrium state. An alternative to the derivation of the fractional kinetic equation (FKE) given by Zaslavsky [24] is presented by West and Grigolini [25]. In this section we present the overlapping highlights of these two derivations in schematic form, emphasizing the physical interpretation.

### 2.1. Generalizing Kinetic Theory

We sketch Zaslavsky’s arguments leading to the FKT resulting from the underlying dynamics being chaotic and consequently the dynamic trajectories being fractal. We begin with the chain condition of Bachelier, Smoluchowsky, Chapman, and Kolmogorov (BSCK) [26]:

(4) $$P\left(x,t\left|x_{0},t_{0}\right.\right)=\int P\left(x,t\left|x^{\prime },t^{\prime }\right.\right)P\left(x^{\prime },t^{\prime }\left|x_{0},t_{0}\right.\right)dy,$$

where $P(x,t\left|x^{\prime },t^{\prime }\right.)$ is the probability density of having a particle at position *x* at time *t* if at time $t^{\prime }\leq t$ the particle was at the point $x^{\prime }$. We make the assumption that the PDF is stationary such that

(5) $$P\left(x,t\left|x_{0},t_{0}\right.\right)=P\left(x,x_{0};t-t_{0}\right),$$

corresponding to the regular scheme for the kinetic derivation [26] and with $\Delta t\equiv t-t_{0}$ we have for the initial condition

(6) $$\lim_{\Delta t\to 0}P\left(x,x_{0};\Delta t\right)=\delta \left(x-x_{0}\right).$$

The first generalization of the historical kinetic theory argument is made by taking into account the fractal nature of the set generated by the ensemble of chaotic trajectories initiated by an underlying non-integrable Hamiltonian. Inserting the time limit for a fractional time differential into the BSCK chain condition enables us to write

(7) $$\partial_{t}^{\alpha }\left[P(x,t)\right]=\lim_{\Delta t\to 0}\frac{1}{\Delta t^{\alpha }}\int dyP\left(x,y;\Delta t\right)-\delta \left(x-y\right)]P\left(y;t\right).$$

This expression can be simplified using a second generalization, that being introducing the generalized Taylor expansion

(8) $$P\left(x,y;\Delta t\right)=\delta \left(x-y\right)+A_{1}\left(y;\Delta t\right)\delta^{\left(\beta \right)}\left(x-y\right)+A_{2}\left(y;\Delta t\right)\delta^{\left(\beta +1\right)}\left(x-y\right),$$

for a set characterized by the fractal dimension 0<β≤1. Inserting this expansion into Equation (7) simplifies the generalized BSCK chain condition by introducing the quantities

(9) $$\begin{array}{ccc} A(x) & \equiv & \lim_{\Delta t\to 0}\frac{A_{1}\left(x;\Delta t\right)}{\Delta t^{\alpha }}=\lim_{\Delta t\to 0}\int dy\frac{{\left|x-y\right|}^{\beta }}{\Delta t^{\alpha }}P\left(x,y;\Delta t\right), \end{array}$$

(10) $$\begin{array}{ccc} B(x) & \equiv & \lim_{\Delta t\to 0}\frac{A_{2}\left(x;\Delta t\right)}{\Delta t^{\alpha }}=\lim_{\Delta t\to 0}\int dy\frac{{\left|x-y\right|}^{\beta +1}}{\Delta t^{\alpha }}P\left(x,y;\Delta t\right). \end{array}$$

Zaslavsky [15] explained that the limit in these two expressions are the result of the fractal dimensionality of the space-time set along which the state of the system is meandering in the Δt→0 limit.

We do not reproduce the mathematical details from the open literature and instead jump to the result for the one-dimensional Fractional Kinetic equation (FKE) [15,25] and write the fractional Fokker–Planck equation (FFPE):

(11) $$\partial_{t}^{\alpha }\left[P(x,t)\right]=\partial_{\left|x\right|}^{\beta }\left[A(x)P(x,t)\right]+\partial_{\left|x\right|}^{\beta +1}\left[B(x)P(x,t)\right].$$

The FFPE has fractional indices in the domain 0<α,β≤1, the fractional time derivative is of the Caputo form, and the fractional spatial derivative is of the symmetric Reisz–Feller form.

So how different are the solutions to the above FFPE from those of the ordinary FPE even when β=1?

### 2.2. Solution to a Simple FKE

One of the simplest dynamical processes described by the FFPE having far-reaching implications has a constant fractional diffusion coefficient and a vanishing fractional velocity:

(12) $$A\left(x\right)=0\;\mathrm{and}\;B\left(x\right)=K_{\beta },$$

thereby reducing Equation (11) to

(13) $$\partial_{t}^{\alpha }\left[P(x,t)\right]=K_{\beta }\partial_{\left|x\right|}^{\beta +1}\left[P(x,t)\right].$$

This is one of the simplest form of anomalous diffusion, first discussed in terms of the continuous time random walk (CTRW) by Montroll and Scher [27]. 

The solution to this fractional diffusion equation is readily obtained by taking its combined Fourier–Laplace transform and introducing the notation

(14) $$F\left\{\partial_{\left|x\right|}^{\beta +1}\left[f(x)\right];k\right\}=-{\left|k\right|}^{\beta +1}\tilde{f}\left(k\right),$$

where $\tilde{f}\left(k\right)$ is the Fourier transform of f(x) and correspondingly

(15) $$L\left\{\partial_{t}^{\alpha }\left[g(t)\right];u\right\}=u^{\alpha }\hat{g}\left(u\right)-u^{\alpha -1}g\left(0\right)$$

where $\hat{g}\left(u\right)$ is the Laplace transform of g(t). Note that in Equation (14) we used the Fourier transform of the Reisz–Feller derivative in space and in Equation (15) we used the Laplace transform of the Caputo derivative in time. Consequently we obtain from the Fourier–Laplace transform of the FFPE:

(16) $$u^{\alpha }P^{*}\left(k,u\right)-u^{\alpha -1}\tilde{P}\left(k,t=0\right)=-K_{\beta }{\left|k\right|}^{\beta +1}P^{*}\left(k,u\right),$$

where the asterisk denotes the double transform of the PDF and the indices lie in the interval 0<α,β≤1. This equation is simplified for the initial value problem:

(17) $$P\left(x,t=0\right)=\delta \left(x\right)\;\Rightarrow \;\tilde{P}\left(k,t=0\right)=1,$$

to the form

(18) $$P^{*}\left(k,u\right)=\frac{u^{\alpha -1}}{u^{\alpha }+K_{\beta }{\left|k\right|}^{\beta +1}}.$$

The inverse Fourier–Laplace transform of this expression yields the solution to the initial value problem for the PDF.

Metzler and Klafter [28] derived the FFPE using the CTRW formalism of Montroll and Weiss [29] and reviewed the potential functions for various combinations of indices. It has also been derived using subordination theory by West [30]. The inverse Laplace transform of $P^{*}\left(k,u\right)$ yields the characteristic function

(19) $$\tilde{P}\left(k,t\right)=E_{\alpha }\left(-K_{\beta }{\left|k\right|}^{\beta +1}t^{\alpha }\right)$$

expressed in terms of the Mittag–Leffler function (MLF):

(20) $$E_{\alpha }\left(z\right)=\underset{n=0}{\sum^{\infty }}\frac{z^{n\alpha }}{\Gamma \left(n\alpha +1\right)}.$$

The inverse Fourier transform of the characteristic function yields the PDF solution

(21) $$P\left(x,t\right)=F^{-1}\left[E_{\alpha }\left(-K_{\beta }{\left|k\right|}^{\beta +1}t^{\alpha }\right);x\right].$$

The simple substitution $k^{\prime }=kt^{\delta }$ into Equation (21), with $\delta =\frac{\alpha }{\beta +1},$ after some algebra reduces the formal solution to

(22) $$P\left(x,t\right)=\frac{1}{t^{\delta }}F^{-1}\left[E_{\alpha }\left(-K_{\beta }{\left|k^{\prime }\right|}^{\beta +1}\right);\frac{x}{t^{\delta }}\right],$$

or in a more familiar scaling form:

(23) $$P\left(x,t\right)=\frac{1}{t^{\delta }}F\left(\frac{x}{t^{\delta }}\right),$$

where the new function is defined:

(24) $$F\left(\frac{x}{t^{\delta }}\right)\equiv P\left(\frac{x}{t^{\delta }},1\right).$$

The function F(·) is analytic in the scaled variable $x/t^{\delta }$, is properly normalized and can therefore be treated as a PDF. For a standard diffusion process, α=1, in which case the MLF becomes an exponential so that for β=1 the Fourier transform can be carried out and this function becomes a Gaussian with δ=1/2. when α=1≠β the result is a stable Lévy process [26,31] with the Lévy index given by 0<1/δ≤2. However, for general chaotic systems there is a broad class of distributions for which the functional form is neither Gaussian nor Lévy.

Mainardi et al. [32] obtained a variety of other solutions to the FKE in terms of the properties of the MLF for 0<α<1. The inverse Fourier transform of the scaled PDF solution for β=1 asymptotically relaxes as the IPL $t^{-\alpha /2}.$

### 2.3. Self-Similar Random Walks

Zaslavsky et al. [33] worked to visualize the underlying landscape produced by averaging over chaotic trajectories and to describe the formal structure uncovered by extensive numerical calculations. They discuss the notion of a “stochastic web” to characterize the chaotic dynamics generated by Hamiltionian systems in which “weak” chaotic orbits are concentrated on small measure domains of phase space thereby constituting a “web”. They note that transport through stochastic webs could produce non-Gaussian, i.e., intrinsically anomalous, diffusion.

The nexus points of the web constitute traps were homoclinic points have dissolved into a spray of local points that locally entrap trajectories for IPL lengths of time. Exiting a trap the orbit undergoes a long–range flight having self-similar properties. The process can be realized as passing through the turnstiles of “cantori” [34]. This argument is realized by replacing the complete simulation of the Hamiltonian dynamics with a random walk (RW) containing the appropriate qualitative features. They do this by way of example whereby they construct a RW determined by a Weierstrass (W) function [35]. Consider the discrete probability described by the stepping PDF for the Weierstrass random walk (WRW) on a one-dimensional lattice with sites indexed by *x* [35]:

(25) $$p\left(x\right)=\frac{a-1}{2a}\underset{n=0}{\sum^{\infty }}\frac{1}{a^{n}}\left[\delta_{x,b^{n}}+\delta_{x,-b^{n}}\right],$$

where *a* and *b* are dimensionless constants greater than one. and $\delta_{ij}$ is the Kronecker delta function: $\delta_{ij}=1$ for i=j and $\delta_{ij}=0$ for i≠j. We follow the analysis of this discrete process given by West and Grigolini [6]. The first notable property of the PDF generated by the WRW is that the second moment of this RW process diverges:

(26) $$\langle x^{2}\rangle =\frac{a-1}{a}\underset{n=0}{\sum^{\infty }}{\left(\frac{b^{2}}{a}\right)}^{n},$$

for $b^{2}>a$ as the series is infinite. The discrete Fourier transform of the PDF given by Equation (25) yields the discrete characteristic function

(27) $$\hat{p}\left(k\right)=\frac{a-1}{a}\underset{n=0}{\sum^{\infty }}\frac{1}{a^{n}}\cos\left[b^{n}k\right].$$

This series was introduced by Weierstrass in 1872 in response to Cantor, a former student and subsequent colleague, who challenged him to construct an analytic function that is continuous everywhere but is nowhere differentiable. Thanks to Mandelbrot [8] we now know that this was the first consciously constructed fractal function and the divergence of the second moment is a consequence of its non-analytic properties.

**Figure 3:**
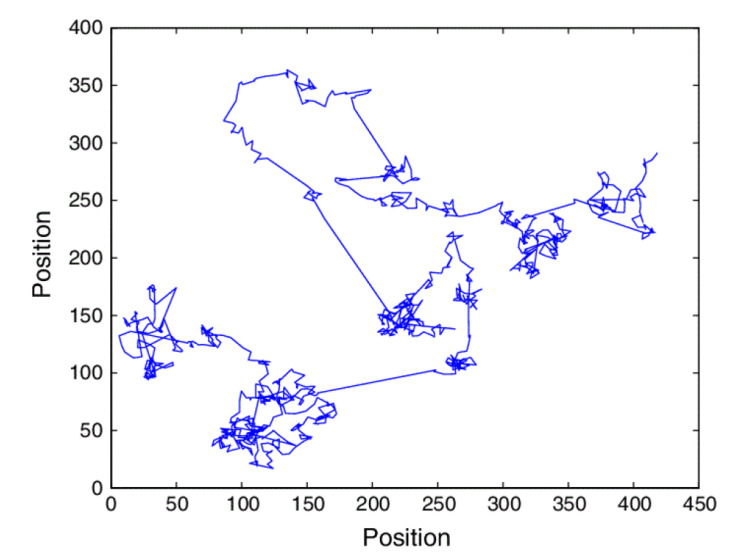
The landing sites for the WRW are depicted and the islands of clusters discussed in the text are readily seen.

As the WRW process unfolds the set of sites visited mimics the influence of localized chaotic islands, interspersed by gaps, nested within clusters of clumps over ever-larger spatial scales. The WRW generates a hierarchy of traps that are statistically self-similar, as suggested by Figure 3. The parameter *a* determines the number of subclusters within a cluster and the parameter *b* determines the scale size between clusters.

The Weierstrass form of the characteristic function allows for a renormalization group (RG) solution [36] from which we can determine the scaling properties of the WRW. Scaling the argument of the characteristic function by *b* and reordering terms in the series allows us to write [33,36]

(28) $$\hat{p}\left(bk\right)=a\hat{p}\left(k\right)-\frac{a-1}{a}\cos k.$$

The RG solution to Equation (28) can be separated into a homogeneous part and a singular part:

(29) $$\hat{p}\left(k\right)={\hat{p}}_{s}\left(k\right)+{\hat{p}}_{h}\left(k\right),$$

where ${\hat{p}}_{h}\left(k\right)$ is analytic in the neighborhood k=0 and ${\hat{p}}_{s}\left(k\right)$ is singular in this neighborhood. The singular part ${\hat{p}}_{s}\left(k\right)$ is obtained by solving the scaling equation:

(30) $${\hat{p}}_{s}\left(bk\right)=a{\hat{p}}_{s}\left(k\right),$$

where we assume the formal solution:

(31) $${\hat{p}}_{s}\left(k\right)=A\left(k\right)k^{\delta }.$$

Inserting this form of the singular solution into Equation (30) yields

(32) $$A\left(bk\right)b^{\delta }k^{\delta }=aA\left(k\right)k^{\delta },$$

providing the distinct equalities

(33) $$\begin{array}{ccc} b^{\delta } & = & a, \end{array}$$

(34) $$\begin{array}{ccc} A(bk) & = & A(k). \end{array}$$

The first equality yields for the power index in terms of the series parameters δ=lna/lnb. The second equality implies that A(k) is periodic in the logarithm of *k* with period lnb. Consequently, the singular part of the RG solution is written

(35) $${\hat{p}}_{s}\left(k\right)=\underset{n=-\infty }{\sum^{\infty }}A_{n}{\left|k\right|}^{H_{n}},$$

with the complex power–law index:

(36) $$H_{n}=\delta +in\frac{2\pi }{\ln b}=\frac{\ln a}{\ln b}+in\frac{2\pi }{\ln b}.$$

The analytic forms of the Fourier coefficients in Equation (35) are given in [35].

Hughes et al. [35] prove that the dominant behavior of the WRW is determined by the lowest-order term in the singular part of the solution for the discrete characteristic function, but we do not show that here. Instead we assume that the dominant behavior is given by the n=0 term in the series:

(37) $${\hat{p}}_{s}\left(k\right)\approx A_{0}{\left|k\right|}^{\delta },$$

whose inverse Fourier transform is determined by a Tauberian theorem to be the IPL:

(38) $$p\left(x\right)=\frac{K(\mu )}{{\left|x\right|}^{\delta +1}},$$

and K(δ) is a known function of δ. Thus, the singular part of the WRW has an IPL stepping PDF and this dominant behavior intuitively justifies ignoring all the other terms in the series.

We now write for the asymptotic time-dependent form of the discrete PDF resulting from the WRW:

(39) $$\begin{array}{ccc} P(x,n+1) & = & \sum_{x^{\prime }}p\left(x-x^{\prime }\right)P\left(x^{\prime },n\right)\\ & = & \sum_{x^{\prime }}\frac{K\left(\mu \right)}{{\left|x-x^{\prime }\right|}^{\delta +1}}P\left(x^{\prime },n\right), \end{array}$$

where we assume that each step *n* in WRW process occurs at equal time intervals. Equation (39) was analyzed in 1970 by Gillis and Weiss [37], who determined that its solution is a Lévy PDF, thereby connecting the RG solution of the WRW to our discussion of the fractional diffusion equation given earlier.

Stable Lévy processes can therefore arise from the “weak” chaotic nature of the phase space trajectories. This is, in part, a consequence of the asymptotic behavior k→0 corresponding to the asymptotic x→∞, which is of significance in determining the transport behavior of the anomalous diffusion process.

## 3. Patterns and Complexity

In the Introduction we identified one of those patterns that is not restricted to a particular discipline, but pops up in every discipline from anatomy to zoology, and that pattern is an allometry relation (AR). However, what distinguishes such patterns from, for example, simple periodic motion? Of course, the existence of such regularity, the pattern of reproducibility in space and time, is what motivated the first investigators to seek common causes to associate with those patterns. Periodic motions, such as vibrations, motivated Hook to introduce his law using Newton’s mechanical force for its explanation. The amazing success of such laws reinforced the idea that other phenomena including the beating of the heart, walking, and the propagation of light could all be described by adopting a similar modeling strategy. However, the *luminiferous aether* is now a quaint historical myth concerning the assumed need for a medium with remarkable properties to support the propagation of electromagnetic waves. In addition, the *normal sinus rhythm* of the heart is a medical myth as heartbeats are not sinusoidal. The more complex the phenomenon being considered the less well the patterns are reproduced using Newton’s view of science.

Much of the present discussion stems from the need to replace Newton’s atavistic characterization of space and time, because they fail to capture the rich structure of the complexity of the modern world. The failure to systematically reexamine these fundamental assumptions have restricted the utility of the modeling techniques of modern physics in the study of the psychology, sociology and the life sciences. The experience of space and time differs between those of the claustrophobic or agoraphobic, from the performer on the stage or the surgeon operating on the brain, from the warrior on the battlefield to the physician on the critical care ward. We require a mathematics that can capture all of this and so much more. The conclusions drawn herein were anticipated a couple of years ago [38]:

What is becoming increasingly clear ... is that the technical intensity of the world has become so dense that the mathematical language initiated by Newton is no longer adequate for its understanding. In fact we now find that we have been looking at the world through a lens that often suppresses the most important aspects of phenomena, most of which are not “simple”. These are characteristics of the phenomena that cannot be described using differential equations and we refer to them as complex.

### 3.1. Allometry through Complexity

We have argued elsewhere [20,39] that the empirical AR given by Equation (1) is a consequence of the imbalance between the complexity associated with the system functionality and the complexity associated with the system size, both being measured by Shannon information. We refer to this as the allometry/information hypothesis (A/I–H) [40] and postulate that in a complex network, composed of two or more interacting subnetworks, the flow of information is driven by the complexity gradient between the subnetworks, transported from that with the greater to that with the lesser complexity.

Implicit in the A/I–H is the assumed existence of dependencies of both system size and system functionality on complexity. Such dependencies have been observed in the positive feedback between social complexity and the size of human social groups [41,42], as well as in ant colony size [43], and the increase in biological complexity with ecosystem size [44]. Other relations have been observed in multiple disciplines, including the increase of prey refuge from predators with habitat complexity [45], computational complexity increasing with program size [46], and gene functionality depending on system complexity [47]. We abstract from these observations that the complexity of a phenomenon increases with system size and that the system functionality increases with system complexity.

The argument presented in this section follows that given recently by West et al. [48] in their discussion of the evolution of military technology over the past millennium. It is intuitively understood, but not often explicitly stated, that size and complexity grow together and are inextricably intertwined through criticality. Moreover, although tied together, their changes are not in direct proportion to one another. A similar connection exists between complexity and system functionality [38]. These interconnections are represented through homogeneous scaling relations, as shown below. West argued that as a system increases in size it provides increasing opportunity for variability, which is necessary in order to maintain stability. Scaling provides a measure of complexity in dynamic systems, indicating that the system’s observables can simultaneously fluctuate over many time and/or space scales. An observable Z(t) scales if for a constant λ it satisfies the homogeneous relation

(40) $$Z\left(\lambda t\right)=\lambda^{\mu_{z}}Z\left(t\right)$$

with the scaling index given by $\mu_{z}$. Note that if we consider the AR given by Equation (1), but without the averaging brackets, the size and functionality depend on a parameter *t*, and scale in the manner indicated by Equation (40), each with a distinct power law index, then $b=\mu_{Y}/\mu_{X}$ in order for the AR to be satisfied.

The hallmarks of fractal statistics are spatial (*z*) inhomogeneity and temporal (*t*) intermittency and the phase space trajectory (z;t) replaces the dynamic variable Z(t). In phase space, the scaling of the dynamic variable is replaced by a scaling of the PDF P(z;t):

(41) $$P\left(z;t\right)=\frac{1}{t^{\mu_{z}}}F_{z}\left(\frac{z}{t^{\mu_{z}}}\right)$$

as given by Equation (23) for general complex phenomena. There is a broad class of PDFs for which the functional form of $F_{z}\left(\cdot \right)$ is left unspecified.

It is straightforward to calculate the average value of Z(t) using the PDF given by Equation (41):

(42) $$\langle Z(t)\rangle =\int zP\left(z,t\right)dz={\bar{q}}_{z}t^{\mu_{z}},$$

and the overall constant is determined by the scaling variable $q=z/t^{\mu_{z}}$ averaged over the PDF F(q):

(43) $${\bar{q}}_{z}\equiv \int qF_{z}\left(q\right)dq.$$

Interpreting Z(t) as the system’s TBM X(t) Equation (42) describes the growth in the overall average size of a complex system with the time *t*, due to the intrinsic dynamics generating increasing complexity. A similar observation can be made interpreting the dynamic variable with a functionality of the system Y(t). Consequently, the same functional form results for both Y(t) and X(t), each with its own index. This is not entirely unexpected since both the functionality and size of the system grow with complexity, but at different rates.

Notice that using the scaling PDF that the average of the dynamic variable now has the scaling property:

(44) $$\langle Z(\lambda t)\rangle =\lambda^{\mu_{z}}\langle Z(t)\rangle .$$

If both the size and functionality of the system can be characterized in terms of the system’s complexity by the same form of scaling PDF we obtain two equations in *t* for the averages. Setting the scaling parameter to λ=1/t, after some algebra we obtain the equalities

(45) $$t={\left(\frac{\langle Y(t)\rangle }{\langle Y(1)\rangle }\right)}^{\frac{1}{\mu_{Y}}}={\left(\frac{\langle X(t)\rangle }{\langle X(1)\rangle }\right)}^{\frac{1}{\mu_{X}}},$$

which can rewritten in the form of the empirical AR given by Equation (1):

(46) $$\langle Y\rangle =a{\langle X\rangle }^{b},$$

with the allometry parameters:

(47) $$a=\frac{\langle Y(1)\rangle }{{\langle X(1)\rangle }^{b}}=\frac{{\bar{q}}_{Y}}{{\bar{q}}_{X}^{b}}\;\mathrm{and}\;b=\frac{\mu_{Y}}{\mu_{X}}.$$

Here, we have used Equation (42) to obtain the second equality for the allometry coefficient. Thus, demonstrating that the empirical AR is the result of the self-similar behavior of the PDF.

Note that the allometry index *b* is expressed as the ratio of $\mu =\alpha /\left(\beta +1\right)$ for the system functionality to that for the system size. In general, this ratio is less than one for both the system size and functionality. It is also the case that for physiological systems b<1. The more the index for the fractional time derivative deviates downward from one, the greater influence the complexity history has on the present behavior of the independent variable, whether functionality or size. The more the index of the fractional variate derivative deviates downward from two, the greater is the nonlocal coupling of the independent variables (functionality or size) across scales. However, these two mechanisms do not independently determine the scaled PDF. It is their ratio that determines the balancing of effects in the functionality and size separately, and then through their ratio to obtain *b*.

It is this coupling across scales in size as well as in physiologic time that entails the temporal AR with b<1, as well as, the positive growth of entropy in approaching the steady state asymptotically. The results of these brief arguments are encapsulated in the Principle of Complexity Management (PCM), which establishes that in the interaction between two complex networks, information flows from the more complex to the less complex network. Information transfer is maximum when the complexities of the two networks are matched [38]. In the time-size application of this section, the PCM takes the form *The origin of natural patterns manifest by temporal ARs is the imbalance between the complexity associated with a system’s measure of time and the complexity associated with a system’s size. In both networks the complexity is measured by the Wiener/Shannon entropy*.

### 3.2. Its about Time

The fundamental question addressed in this section is whether time outside the physical sciences, say the time for a scurrying mouse at the lower left of Figure 1 is the same as that of the lumbering elephant at the upper right of the metabolic AR curve. Newton would assert that they are identical and we would agree that the time shared by the two animals is the same when referenced to an external mechanical clock. However, are the two times the same when referenced to their individual physiological clocks? This question arises because the lifespans of the two creatures are essentially the same when their lifetimes are measured using the product of the number of heartbeats times the average time interval between beats. This is very different from the comparison of their separate lifespans when referenced to an external clock in which case the two differ by years. This change of reference of time measures, from the ticking of a clock to the beating of a heart, suggests that physiological time may be a monotonically decreasing function of physical time [49].

This difference in the meaning of time has lead to such concepts as biological time [50], physiologic time [51], and metabolic time [52], all in an effort to highlight the distinction between time in living and in inanimate systems. The intrinsic time in a living process was first called biological time by Hill [53], who reasoned that since so many properties of an organism change with size that time itself ought to scale with TBM. Natural scientists have subsequently hypothesized that physiologic time differs from the time measured by the ticking of a mechanical clock, or Newtonian time, in that the former changes with the size of the animal [17,18], whereas the latter does not [54].

**Figure 4:**
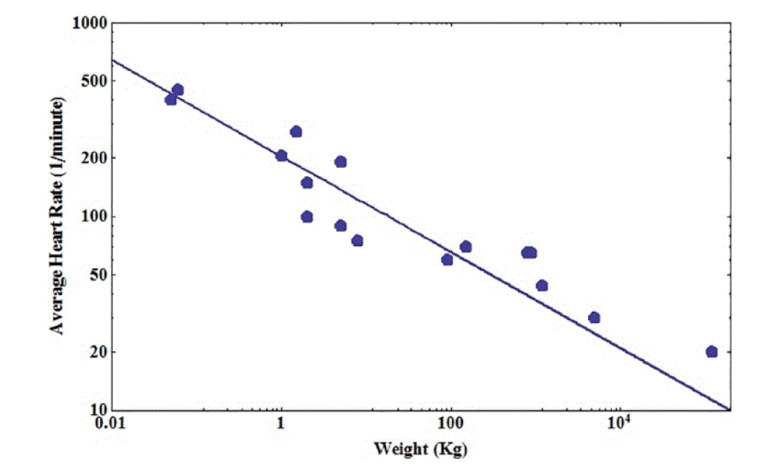
The average heart rate in beats per minute for 16 animals from the fastest, hamsters, to the slowest, large whales, with humans being in the middle of a fitting curve. The data were obtained from [57] and the solid line segment is fitted to the temporal AR. From the work in [49] with permission.

Lindstedt and Calder [55] developed the concept of biological time further and determined experimentally that biological time, such as species longevity, satisfies a temporal AR with the functionality of the system being the physiologic time Y=τ and *X* the TBM *M* [56]:

(48) $$\langle \tau \rangle =a{\langle M\rangle }^{b}$$

which describes the average duration of biological events. In Figure 4, we record the average heart rate $R=1/\langle \tau \rangle$ for sixteen animals [57] covering six orders of magnitude in average TBM. The solid line segment is the fit to the data with empirical values to the allometry parameters given by a=205 and b=0.248, with a quality of fit measured by $r^{2}=0.96$. Other, more exhaustive, fits to larger data sets, made by other investigators, support the notion that physiologic time is extensive and may be found in many other places [17,18], but the results are equivalent.

In an allometry context, one version of the FKE, would be given by Equation (13) where the phase space variables (z,t) are here given by (m,τ) [30] and P(m,τ)dm is the probability that the dynamic mass variable M(τ) lies in the interval (m,m+dm) at time τ. M(τ) represents the TBM of a mature individual species member, within an ensemble of realizations, at the physiological time τ. The exact solution to the FKE has been obtained as the inverse Fourier transform of the characteristic function, expressed in terms of the Mittag–Leffler function given by Equation (21) with the variables properly defined. The allometry coefficient in this temporal AR has a theoretical value expressed in terms of the average of the scaled variable $q=m/\tau^{\delta }.$ Consequently, the complexity of the underlying physiology of an animal entails the physiologic time through the scaling statistics.

The dependence of the empirical AR on the overall state of the system is captured by the entropy. The Wiener/Shannon information entropy associated with the system manifesting temporal allometry has the value

(49) $$S\left(\tau \right)=-\int P\left(m,\tau \right)\log_{2}P\left(m,\tau \right)dm$$

which when the scaled PDF given by Equation (41) is inserted into the integral yields

(50) $$S\left(\tau \right)=S_{0}+\delta \log_{2}\tau$$

where $S_{0}$ is the entropy referenced to the PDF F(·).

Consequently, as we mentioned earlier, given a monotonic function relating physical and physiologic time t=g(τ), such that

(51) $$\frac{dg(\tau )}{d\tau }\equiv \;\overset{\cdot }{g}\;\geq 0$$

we have for the physical time derivative of the entropy Equation (50):

(52) $$\frac{dS(\tau )}{dt}=\frac{\delta }{\tau }\frac{1}{\overset{\cdot }{g}}\geq 0$$

Consequently, the entropy generation in physical time for the physiologic process entailing the temporal AR is positive semidefinite. Thus, the rate of entropy generation in Newtonian time is consistent with the dynamics of living systems having their own physiological time.

It is worth pointing out that empirical ARs are not necessarily restricted to living systems, but also arise in social systems as well. This is not entirely unexpected, sa the average mass in an empirical AR is actually a surrogate for the living system’s complexity. Proceeding by analogy, one might anticipate that such an AR should appear in a social context, where the average TBM is replaced with the average population or population density. This does, in fact, occur in the form of ARs where the functionality is expressed in terms of the rate at which an event occurs. An exemplar is Farr’s Law, which dates back to the nineteenth century, and quantifies the “evil effects of crowding”, relating a population’s mortality rate to an institution’s patient population density in the form of a rate AR [38,58]. Other examples of social ARs include an increasing urban crime rates, the more rapid spread of infectious diseases, and a speedup in pedestrian walking, all with increasing city size, as quantitatively confirmed by Bettencourt et al. [59]. Unlike the biological case, in the social rate ARs the allometry index has a value greater than one, b>1, confirming that cities have, at all times and in all places, throughout history, entailed increased rates in human activity, for good or ill.

## 4. Subordination

The Montroll–Weiss (MW) perspective of CTRW [29] has been used to support the assumption that there are at least two distinct, but related, interpretations of time associated with a system’s dynamics. As noted in the Introduction, the first is the external time associated with an objective observer who records the behavior of the system. This is Newton’s assumption of what constitutes time: it is experimental or clock time. The second kind of time is the local time associated with the internal dynamics of the system, called subjective or operational time. In a psychological experiment the latter time is what is experienced by the participant. The experimental observation, carried out in the clock time *t*, is subordinated to a process occurring in the operational time *n*. For simplicity, we assume the operational time *n* to be an integer number so large as to become indistinguishable from a continuous variable. In the operational time *n* the evolution of the PDF describing the process is described by the ordinary diffusion equation

(53) $$\frac{\partial P(x,n)}{\partial n}=D\frac{\partial^{2}P\left(x,n\right)}{\partial x^{2}}=LP\left(x,n\right),$$

where $L\equiv D\frac{\partial^{2}}{\partial x^{2}}$ is the diffusion operator.

The dynamics generating the diffusion process is the simple Langevin equation

(54) $$\frac{dX(n)}{dn}=\eta \left(n\right),$$

where X(n) is the space coordinate at time *n* and $\eta \left(n\right)$ is the fluctuating velocity. If the velocity is a stochastic process with delta correlated fluctuations, this equation yields a diffusion process with scaling index δ=1/2. If δ≠1/2 the diffusion is anomalous and is the result of memory influencing the fluctuations. In the present representation η(n) of Equation (54) is totally random, i.e., it has no memory. However, in the clock time, the event η(n) occurs at time t(n) and the independent event η(n+1) at time t(n+1) with the time distance τ(n)=t(n+1)−t(n) derived from a waiting time PDF ψ(τ). We are interested in the case where the waiting time PDF has the hyperbolic form:

(55) $$\psi \left(\tau \right)=\left(\mu -1\right)\frac{T^{\mu -1}}{{\left(T+\tau \right)}^{\mu }}$$

We use this hyperbolic form to define the concept of crucial event.

Crucial events are defined by the time interval separating the occurrence of consecutive events. The time intervals between crucial events are determined by a waiting time PDF with the same time asymptotic behavior as Equation (55), with the condition 1<μ<3. In clock time we use the theoretical MW prescription [29] to obtain

(56) $$P\left(x,t\right)=\underset{n=0}{\sum^{\infty }}\overset{t}{\int_{0}}dt^{\prime }\psi_{n}\left(t^{\prime }\right)\Psi \left(t-t^{\prime }\right)e^{nL}P\left(x,0\right).$$

Note that $\psi_{n}\left(t^{\prime }\right)$ is the PDF that *n* events have occurred and that the last event took place at time $t^{\prime }$.

For the formula given by Equation (56) to hold with *n* going to *∞*, we must assume that for the random walker to travel the distance *x* in a time *t* a virtual infinitely large number of events may occur, thereby implying the diffusion coefficient *D* is extremely small. In the case μ<2, the mean waiting time $\langle \tau \rangle$ diverges, thereby providing an additional reason for the experimental observation time *t* to be large.

It is possible to prove, using the arguments developed by Allegrini et al. [60] with a minor notational change, that Equation (56) is equivalent to the integro-differential phase space equation:

(57) $$\frac{\partial P(x,t)}{\partial t}=\underset{0}{\int^{t}}dt^{\prime }\Phi \left(t-t^{\prime }\right)LP\left(x,t^{\prime }\right),$$

where Φ(t) is the MW memory kernel related to the waiting–time PDF and $\psi \left(t\right)=\psi_{n=1}\left(t\right)$. In the Laplace transform representation where $\hat{f}\left(u\right)$ denotes the Laplace transform of f(t), this latter relation is

(58) $$\hat{\Phi }\left(u\right)=\frac{u\hat{\psi }\left(u\right)}{1-\hat{\psi }\left(u\right)}.$$

In the case where the index for the hyperbolic PDF, which asymptotically is the IPL index, is in the interval 1<μ<2, using Equation (58) it is shown [61] that asymptotically u→0:

(59) $$\hat{\Phi }\left(u\right)\approx u^{1-\alpha }.$$

Inserting this asymptotic expression into the Laplace transform of Equation (57) and taking the inverse Laplace transform yields the fractional diffusion equation (FDE):

(60) $$\frac{\partial^{\alpha }P\left(x,t\right)}{\partial t^{\alpha }}=LP\left(x,t\right)$$

Here, the fractional time derivative is of the Caputo form with α=μ−1<1. We note here that the analytic solution to Equation (60) is given by the scaling PDF Equation (23) when β=1 and δ=α/2.

Culbreth et al. [62] stress certain subtleties of these formal results to provide a context with which to appreciate their contribution to the field of cognition and to the fractional calculus. First, they notice that we can use psychological arguments to interpret the connection between operational time and clock time, as done in [63]. The operational time is subjective in this psychological context with a logarithmic connection with the clock time *t*, which changes an exponential waiting time PDF into the hyperbolic structure of Equation (55). This property provides the rationale for why they [62] consider the CTRW formalism to be closely connected to the issue of cognition. As they point out, earlier work [60] analyzed a series of events using the hyperbolic waiting time PDF using the Kolmogorov–Sinai definition of complexity and determined that the signal becomes computationally compressible for 2<μ<3. This is equivalent to assessing that the time series hosts messages that can be decoded.

On the other hand, the Kolmogorov–Sinai entropy vanishes for μ<2 and has been recently generalized to take into account the rare crucial events [64] of this region. These crucial events are conjectured to be the signal of swarm intelligence [65], while the observation of the dynamics of the brain leads to the conclusion that μ=2 is a proper signature of the brain of an awake subject [66]. In summary, the events characterized by the inter-event or hyperbolic waiting time PDF are considered to be a signature of cognition and are known to be responsible for the transport of information from one intelligent system to another [67,68]. The term crucial events is a proper nomenclature to acknowledge the importance of these rare events.

## 5. Discussion and Conclusions

We began this essay with the stated intent of supporting the Complexity Hypothesis by demonstrating to the reader why Newton’s dynamic view of physical objects is not just inappropriate for living and social systems but its domain of application within the physical sciences is shrinking dramatically as well. The unexamined assumptions regarding the nature of space and time, with which Newton opened his *Principles*, make his force law invalid for the study of complex phenomena. Yet, these are the phenomena of interest to scientists in the 21st century, whether such phenomena reside in the physical, social, or life sciences.

As mentioned, Newton’s equations have been shown to require changes when particles are moving very fast (approaching the speed of light), when the spatial scales are very large (cosmological) and when they are very small (quantum mechanical). In each of these domains the dynamic laws follow a correspondence principle in that they converge on Newton’s laws by changing a parameter value to replicate the world of our five senses. Herein we have shown that in this world of experience we continually encounter deviations from Newton’s laws at normal speeds and spatial scales, due to chaos. Chaotic dynamics led to replacement of the probability calculus of Kinetic Theory with that of FKT, as well as to operational time. One way to measure the degree of complexity generated by chaotic attractors is by using the entropy of the behavior.

Crutchfield et al. [69] interpreted the entropy of a dynamic process as the average rate of information generation by a chaotic process in that the more precisely an initial state of a system is specified, the more information one has available. The amount of information contained in the initial state is inversely proportional to the state space volume $V_{i}$ localized by measurement. Trajectories initiated in a local volume of a regular attractor remain close to one another as the system evolves, and therefore no new information is generated, while the initial information is preserved in time. Consequently, the initial information can be used to predict the system’s final state.

On the other hand, on a chaotic attractor the initial volume gets smeared out, consequently, as the system evolves the initial information is destroyed and replaced by newly created information. Thus, the volume in the specification of the initial system is eventually spread over the entire attractor and all predictive power is lost since the probability of being anywhere on the attractor is the same. All causal connection between the present state of the system and its future or final state is lost. This is referred to as the sensitive dependence on initial conditions.

Let us denote the final region of phase space the system occupies by $V_{f}$ so that the change in the observable information ΔI is determined by the volume change from the initial to final state [70,71]:

(61) $$\Delta I=\log_{2}\left(\frac{V_{f}}{V_{i}}\right).$$

The time rate of information change (creation or dissipation) is therefore

(62) $$\frac{dI}{dt}=\frac{1}{V}\frac{dV}{dt},$$

where the time-dependent volume *V* over which the initial conditions are spread determines the ultimate fate of the initial information. In regular, which is to say non-chaotic, systems the sensitivity of the flow in the initial conditions grows with time no more rapidly than a polynomial. Let Ω(t) be the number of states at time *t* that can be distinguished such that if the greatest polynomial index is *n* such that $\Omega \left(t\right)\propto t^{n}.$ The ratio of the final to initial volume in such a system is equal to the relative number of states independently of the time $\frac{V_{f}}{V_{i}}=\frac{\Omega_{f}}{\Omega_{i}}$, so that for the rate at which information changes [71]:

(63) $$\frac{dI}{dt}∼\frac{n}{t}.$$

Thus, the rate of generation of new information decreases with time and converges to zero as t→∞. As in Poincaré’s quote in the Introduction, the final state is approximately predictable from the approximate initial information.

On the other hand, in chaotic systems two trajectories separate exponentially and therefore the number of distinguishable states grows exponentially with time $\Omega \left(t\right)\propto \exp\left(\lambda t\right)$, where λ is the Liapunov coefficient. In this case, the rate at which information is generated is constant:

(64) $$\frac{dI}{dt}∼\lambda .$$

In this latter system, information is continuously generated by the attractor independently of the initial state. Nicolis and Tsuda [70] used this property of chaotic dynamic systems in the early modeling of cognitive systems using nonlinear dynamics and subsequently for information processing in neurophysiology, cognitive psychology, and perception [72].

Thus, Newton’s statements about the absolute nature of space is contradicted by the chaotic trajectories entailed by his own force law when applied to complex systems. Subsequently, even Kinetic Theory and the introduction of stochastic differential equations, which were early attempts to make the differential calculus and complex phenomena compatible, could only be salvaged by means of the FC. In a similar way, Newton’s statements regarding the absolute nature of time have been shown to have little place, if any, outside restricted domains of the physical sciences.
